# Coordination of Cilia Movements in Multi-Ciliated Cells

**DOI:** 10.3390/jdb10040047

**Published:** 2022-11-11

**Authors:** Masaki Arata, Fumiko Matsukawa Usami, Toshihiko Fujimori

**Affiliations:** 1 Division of Embryology, National Institute for Basic Biology, 5-1 Higashiyama, Myodaiji-cho, Okazaki 444-8787, Japan; 2 Department of Basic Biology, School of Life Science, SOKENDAI, The Graduate University for Advanced Studies, 5-1 Higashiyama, Myodaiji-cho, Okazaki 444-8787, Japan; 3 Institute of Plant Science and Resources, Okayama University, 2-20-1 Chuo, Kurashiki, Okayama 710-0046, Japan

**Keywords:** cytoskeleton, motile cilia, multi-ciliated cells, planar cell polarity

## Abstract

Multiple motile cilia are formed at the apical surface of multi-ciliated cells in the epithelium of the oviduct or the fallopian tube, the trachea, and the ventricle of the brain. Those cilia beat unidirectionally along the tissue axis, and this provides a driving force for directed movements of ovulated oocytes, mucus, and cerebrospinal fluid in each of these organs. Furthermore, cilia movements show temporal coordination between neighboring cilia. To establish such coordination of cilia movements, cilia need to sense and respond to various cues, including the organ’s orientation and movements of neighboring cilia. In this review, we discuss the mechanisms by which cilia movements of multi-ciliated cells are coordinated, focusing on planar cell polarity and the cytoskeleton, and highlight open questions for future research.

## 1. Introduction

**Figure 1:**
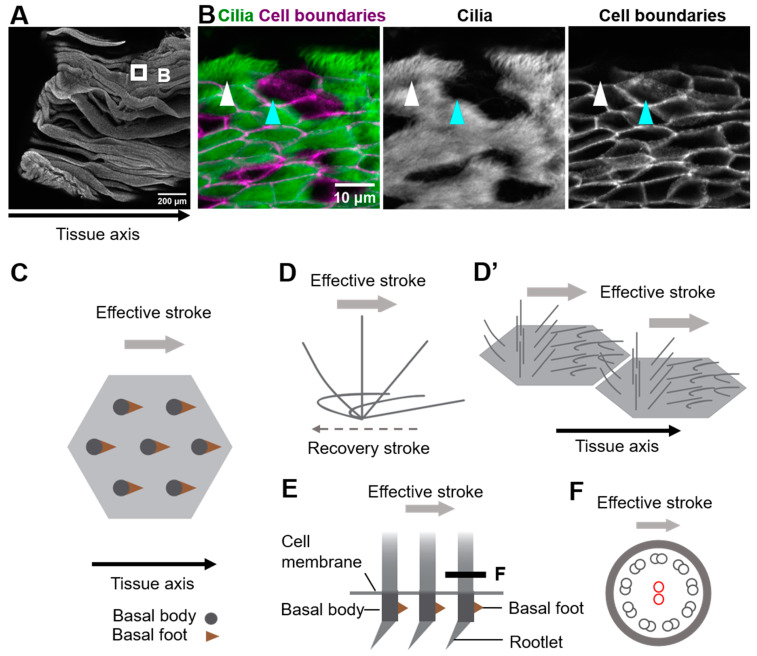
Coordination of cilia movements in multi-ciliated cells. (**A**) Ovary end of the oviduct was opened longitudinally and stained with E-cadherin. (**B**) Cilia (green; acetylated tubulin) and cell boundaries (magenta; E-cadherin) of the oviduct epithelium are visualized. The oviduct epithelium is composed of multi-ciliated cells (white arrowhead) and secretory cells (cyan arrowhead). At the apical surface of those multi-ciliated cells, about 150 cilia are formed on average. (**C**) Representative apical views of multi-ciliated cells. Gray circles and brown triangles indicate basal bodies and basal feet, respectively (also see Figure 1(**E**)). Note that the basal foot points in the same direction as the effective stroke (gray arrow). (**D**) Schematic representation of the movement of an individual cilium. Cilia repeat cyclic movements comprised of a fast effective stroke (gray arrow), and a slow recovery stroke (a backward motion; dotted arrow). (**D’**) A schematic of cilia movements in multi-ciliated cells. Apical surfaces of two multi-ciliated cells are shown. Note that the orientation of effective stroke (or recovery stroke) is consistent with that of the tissue axis. The phase of the beating cycles of cilia shifts between neighboring cilia and generates a metachronal wave, which is a wave-like propagation of cilia movements. (**E**) Lateral view of the basal region of cilia. The basal body has two appendages, the basal foot and the rootlet. (**F**) Cross-sectional view of the cilium along the gray line in (**E**). 9 + 2 microtubules are shown in small circles. The central pair of 9 + 2 microtubules (red circles) run perpendicular to the direction of the effective stroke (gray arrow).

Multi-ciliated cells line the surface of the oviduct, the trachea, and the ventricle of the brain (Figure 1A,B show oviduct multi-ciliated cells). At the apical surface of multi-ciliated cells, tens to hundreds of motile cilia are formed. The directions of cilia movements are unidirectionally aligned within each cell (rotational polarity), and they are consistent with the orientation of the tissue axis (tissue-level polarity). In addition, cilia exhibit a metachronal wave, a wave-like propagation of cilia movements in the plane of the tissue, which is produced by a temporal coordination of cilia movements between neighboring cilia (Figure 1C–D’) [1,2,3,4,5]. The coordination of cilia movements is essential for the functions of organs. In the mammalian oviduct, cilia pick up ovulated oocytes at the ovary end of the oviduct and carry them to the uterus [6,7]; in the trachea, cilia transport mucus and eliminate debris and pathogens [8,9]; and in the ventricle, cilia generate a flow of cerebrospinal fluid that is required for homeostasis of the organ [10].

Motile cilia of multi-ciliated cells are microtubule-based cell protrusions which show a biphasic movement comprised of a fast effective stroke, and a slow backward motion, the recovery stroke (Figure 1D). Inside the majority of motile cilia, 9 + 2 arrays of microtubules run along the longitudinal axis of the cilium, and a central pair of microtubules lie perpendicular to the beating orientation of the cilium (Figure 1E,F) [11]. The basal body lies at the base of cilium, and the basal foot and the rootlet are associated with the basal body (Figure 1E). A single basal foot protrudes from the lateral side of each basal body, and its direction is consistent with that of the effective stroke [12,13]. A rootlet is located at the proximal end of each basal body and extends to the center of the cell [14,15,16,17]. As described in later sections, those basal structures are connected to the cytoskeleton, which is essential for coordinating cilia movements.

To establish a tissue-level coordination of cilia movements, how do cilia know the orientation of the tissue and movements of neighboring cilia, and modulate their orientation and movements? Here, we review recent findings that may answer these questions and offer a discussion based on open questions.

## 2. Roles of Core PCP Proteins in Coordinating Cilia Orientation

**Figure 2:**
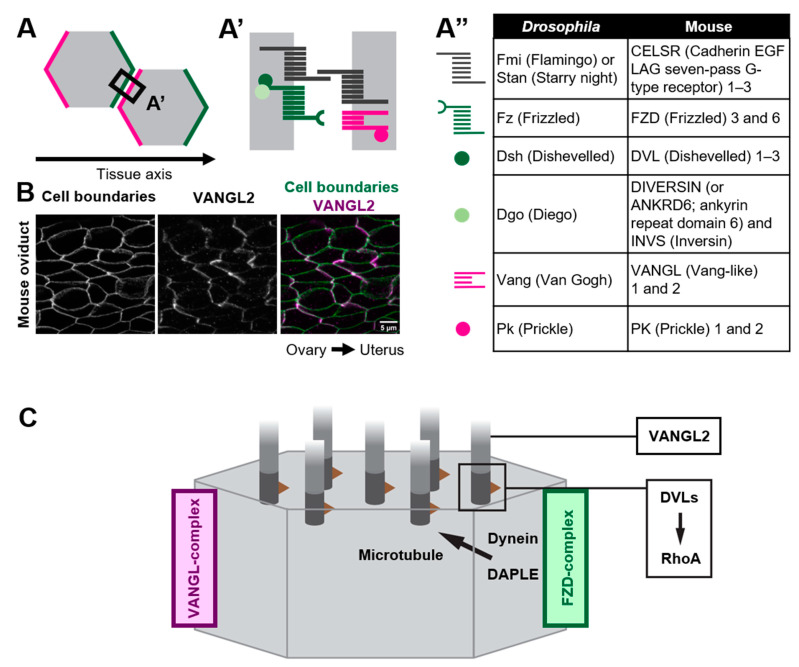
Polarized distribution of core PCP proteins along the tissue axis. (**A**–**A”**) Core PCP proteins form an asymmetric complex at cell boundaries. (**A**,**A’**) FZD-containing complex (FZD-complex; green) and VANGL-containing complex (VANGL-complex; magenta) are segregated to opposite cell boundaries (note that their distribution is polarized along the tissue axis). Extracellular domain of CELSRs provides intercellular bridges between the FZD-complex and the VANGL-complex, thus enabling the coupling of cell polarity at a multicellular level. (**A”**) A list of members of core PCP proteins in *Drosophila* and their counterparts in mice. (**B**) Mouse oviduct epithelium was stained for E-cadherin and a core PCP protein, VANGL2. E-cadherin labels cell boundaries. Note the zigzag pattern of VANGL2 signals which highlights polarity in the cell-boundary distribution of VANGL2 along the ovary–uterus axis. (**C**) Mechanisms by which core PCP proteins control the coordinated movements of cilia.

As exemplified by the unidirectionally beating cilia of multi-ciliated cells, various epithelial cells polarize not only along the apical-basal axis (inside–outside axis) of epithelial tissues, but also on the plane of epithelial tissues. The latter cell polarity, which is perpendicular to the apical–basal axis, is referred to as planar cell polarity (PCP) [18,19,20,21,22,23,24,25,26,27,28]. Pioneering research using the wings of *Drosophila melanogaster* identified a group of proteins, core PCP proteins, that orchestrate the establishment of PCP (Figure 2). Core PCP proteins are an evolutionally conserved group of proteins comprised of transmembrane proteins, Flamingo/Starry night (Fmi/Stan), Van Gogh (Vang) and Frizzled (Fz), as well as cytoplasmic proteins Prickle (Pk), Dishevelled (Dvl) and Diego (Dgo) [21,26,29,30]. Each *Drosophila* wing epithelial cell forms an actin-rich cell protrusion, a wing hair, at the apical cortex and each wing hair points to the distal end of the wing, which is a hallmark of PCP in the wing [21]. Just before the onset of wing hair formation, cell boundary localization of core PCP proteins is strongly biased along the tissue axis (Figure 2A). Fz- and Vang-containing complexes (referred to as the Fz- and Vang-complex in *Drosophila*, respectively, or the FZD- and VANGL-complex in vertebrates, as used hereafter) localize at the distal and proximal side of the cell, respectively (Figure 2A,A’) [28,31,32,33,34,35]. When each member of core PCP proteins is lacking, the orientations of wing hairs are not coordinated along the tissue axis [21]. In addition to the *Drosophila* wing, the asymmetric distribution of core PCP proteins was observed in various organs and animals, including multi-ciliated cells of the mouse oviduct, trachea, and ventricle (Figure 2B) [20,36,37,38]. The establishment of the polarized distribution of core PCP proteins precedes the formation of multi-cilia in the developing oviduct [20,39]. Furthermore, the loss of core PCP proteins abrogates the orientation of cilia, suggesting that the asymmetric distribution of core PCP proteins provides a cue to orient cilia. In contrast to *Drosophila* genes encoding core PCP proteins, those in vertebrates are often duplicated and might have divergent functions (Figure 2A”). For example, there are three homologs of *Drosophila* Flamingo, CELSR1, 2, and 3 in mice, and these have different expression patterns and show different phenotypes when their functions are lost [20,40,41,42]. Here, we review general and some variable roles of core PCP proteins in coordinating cilia orientation in multi-ciliated cells.

### 2.1. Multicellular and Tissue-Level Coordination of Cilia Orientation

An important role of core PCP proteins in PCP establishment is their non-cell-autonomous effect on the orientation of adjacent cells [23]. CELSRs are atypical cadherins and their homophilic interactions through their extracellular domains enable the intercellular coupling of FZD- and VANGL-complexes (Figure 2A,A’) [23]. In *Xenopus* skin, the transplantation of *VANGL2*-overexpressing tissues showed non-cell autonomous effects on adjacent wild type cells. In these wild type cells, cilia pointed away from the transplant. *VANGL2*-knocked down transplants showed opposite effects to the overexpressing transplants. In other words, cilia always pointed to cells with lower VANGL2-level [43]. In the oviduct of *CELSR1* knockout mice, cilia orientation is still coordinated in each cell, but the mean angle of cilia orientations in each cell varies among adjacent cells [39]. These observations suggest that core PCP proteins are required for the intercellular coordination of cilia orientation [41].

To align the orientation of cells along the tissue axis, factors that transmit information regarding tissue orientation are necessary. Such factors are referred to as global factors, which include a gradient of extracellular concentration of WNT molecules [44,45,46], differences in the level of expression of atypical cadherins Dachsous/Fat and their modulator Four-jointed [23,25,47,48,49,50], forces exerted on epithelial tissues [51,52,53,54], and fluid flow [37,55]. Those global cues somehow control the localization of core PCP proteins at cell boundaries. For example, in *Xenopus* skin, core PCP proteins are stabilized at cell boundaries that lie perpendicular to the direction of gastrulation. When mechanical strain was artificially applied to *Xenopus* skin, localization of core PCP proteins was stabilized at cell boundaries, suggesting that the mechanical strain generated by gastrulation acts as a global cue [54]. However, mechanisms by which global cues control the localization of core PCP proteins, and what acts as a global cue in each organ, are still largely unknown.

### 2.2. How Do Core PCP Proteins Orient Cilia?

Various forms of evidence suggest that the distribution of core PCP proteins at the cell boundary provides a directional cue for orienting cilia. If so, how do core PCP proteins control cilia that emerge from distant locations? Recent studies suggest that core PCP proteins orient cilia via microtubules [38,56,57,58]. In ependymal cells of the ventricle, a molecular motor dynein is localized at the cell cortex where the FZD-complex is enriched. Dishevelled-associating protein DAPLE is required for the localization of dynein at the cell cortex, and the loss of DAPLE and the inhibition of the activity of dynein both abrogate the orientation of cilia [56,59]. It has been proposed that dynein at the cell cortex pulls microtubules that connect the cell cortex and basal bodies, and this pulling force might orient cilia to FZD-enriched cell boundaries (Figure 2C) [56]. DAPLE is also required for the establishment of rotational polarity in the trachea, although it is unclear whether the role of DAPLE is the same as in ependymal cells. In the trachea, DAPLE binds to FZD6, and bundles and stabilizes nearby microtubules, and this concentrates microtubules around the FZD6-enriched cell cortex [58]. A theoretical analysis incorporating a hydrodynamic interaction between cilia and microtubules suggests that such an asymmetric concentration of microtubules is sufficient to orient cilia [57]. A similar asymmetric concentration of microtubules was reported in the mouse oviduct [39]. In a *CELSR1* mutant oviduct, microtubules were still concentrated in more than 75% of multi-ciliated cells, while the orientation of the concentration was not aligned along the tissue axis. Importantly, the orientation of the concentration was consistent with that of cilia in each *CELSR1* mutant cell [39]. Therefore, CELSR1 control the orientation of the concentration of microtubules, thus aligning cilia along the body axis. These results suggest that core PCP proteins provide directional information to cilia via microtubules that is sensed by basal bodies.

Interestingly, in the *Xenopus* skin, an effective stroke pointed to the direction of VANGL-complex-enriched cell boundaries [60]. This relationship is reversed in the mouse oviduct, trachea, and ventricle, where the direction of recovery stroke and that of VANGL complex-enriched cell boundaries are consistent [37,38,42]. Mechanisms of how core PCP proteins control cilia orientation might be different among tissues and animals.

### 2.3. Variable Roles of Members of Core PCP Proteins in Multi-Ciliated Cells

A simplified view of how core PCP proteins orient cilia is as follows: (1) global cues orient the cell boundary-localization of core PCP proteins along the tissue axis and (2) core PCP proteins orient cilia toward core PCP proteins-enriched cell boundaries via microtubules. However, complexities reside in this core PCP proteins-dependent mechanism. Genetic analyses suggest that each member of core PCP proteins plays different roles in multi-ciliated cells. In the brain ventricle, intercellular coordination of cilia orientation require CELSR1, while rotational polarity depends on CELSR3 and VANGL2 [41].

In addition to cell boundaries, core PCP proteins are also detected at cilia. DVLs are localized at the base of cilia in *Xenopus* skin, mouse trachea and ventricle [37,38,61], and VANGL2 is localized along the cilia in mouse ventricle [37]. DVL1, DVL2, and DVL3 seem to show different subcellar localization in a tissue-dependent manner. Whereas DVL1 and DVL3 are localized at cell boundaries, DVL2 is localized only at the base of cilia in mouse trachea [38]. In the mouse ventricle, DVL1 and DVL2 are detected at the patch of basal bodies [37,62,63]. In *Xenopus* skin, knockdown of *DVLs* disrupted the apical migration of basal bodies, and impacted ciliogenesis. Furthermore, when a deletion form of DVL, Xdd1, was expressed in multi-ciliated cells, ciliogenesis was weakly affected, but the orientation of cilia was no longer aligned in each cell. In addition to Xdd1, misexpression of a dominant negative form of RhoA severely misoriented cilia. Since rGBD, which binds to active RhoA, was concentrated in foci at the apical surface of multi-ciliated cells and those foci were lost when DVLs became depleted, DVLs might control cilia orientation via the activation of RhoA at basal bodies (Figure 2C) [61]. Furthermore, functions of DVL in cilia might be regulated post-transcriptionally. PTEN dephosphorylates serine 143 of DVL2, and the loss of PTEN affects ciliogenesis and/or the polarity of cilia in *Xenopus* skin, and mouse trachea and ventricle [64]. In addition to DVLs, CELSRs also regulate ciliogenesis. In ventricles lacking both CELSR2 and CELSR3, basal bodies do not migrate to the apical surface of cells during the differentiation of multi-ciliated cells [36]. These findings indicate that core PCP proteins function at two different locations, at cilia and at cell boundaries.

## 3. Fluid Flow Orients Cilia

As discussed above, core PCP proteins play key roles in coordinating cilia orientation. In addition, a hydrodynamic effect also controls the direction of ciliary movements. When an artificial fluid flow was applied to *Xenopus* skin, cilia reoriented in accordance with the direction of the flow [55]. Such an effect of fluid on cilia orientation was verified in various tissues, including ependymal cells in the mouse ventricle [37]. In cultured trachea epithelial cells, multicellular coordination of the orientation of ciliary movements has autonomously emerged. This self-organization was suggested to depend on a hydrodynamic coupling between cilia and the overlying mucus layer [65].

Although multi-ciliated cells in various organs respond to fluid flow and change the orientation of cilia movements, the responsiveness of cilia to this flow varies depending on the density of cilia. The response of mature ependymal cells to fluid flow was weaker than that of immature cells [37]. Such a change in the response to this flow depends on the sheltering effect by cilia. The more ependymal cells mature, the greater the density of cilia. As a result, each cilium is sheltered from the fluid, thereby weakening the effect of fluid on cilia [66,67]. Of note, mature ependymal cells do not completely lose their responsiveness to this flow. When they are exposed to a strong flow, they can still change their beating direction. Consistently, in the mouse ventricle, the direction of cilia-generated flows changes periodically [68]. In contrast to ependymal cells where a patch of tens of cilia is formed, hundreds of cilia cover the entire surface of multi-ciliated cells in the oviduct and trachea, implying that the sheltering effect is larger in these organs. A recent study suggested that in the mouse oviduct, fluid flows toward the ovary [69]. In other words, cilia beat in an opposite direction to the fluid flow. In the oviduct, a direct interaction between an egg and cilia may play a key role in the transportation of eggs along the effective strokes of the cilia, and the flow of oviduct fluid is a proposed driving force that transports sperm to the egg [69]. A sheltering effect might partly explain why cilia do not orient along the fluid flow in the oviduct, although it is still unclear whether oviduct cilia are able to respond to the flow and whether the strength of the flow is sufficient to orient cilia.

How, then, do cilia sense the direction of the flow and change their orientation? Ependymal cells lacking VANGL2 do not respond to the flow, although they form structurally normal cilia and the beating activity of cilia remains intact [37]. Several members of core PCP proteins, including VANGL2 and DVLs, localize at cilia in addition to cell boundaries. Those cilia-localized core PCP proteins might be required for orienting cilia in response to the fluid flow (Figure 2C).

## 4. Metachronal Wave: A Temporal Coordination of Cilia Movements

The unidirectional beating of cilia is essential for generating fluid flow. However, if the timing of an effective stroke is not coordinated between adjacent cilia, how is an efficient flow generated? In ciliated organs, such as the trachea, the phase of cilia movement shifts slightly between adjacent cilia, and this results in a propagating-wave-like pattern of cilia movements at the multicellular level. Such a temporal coordination of cilia movements is referred to as a metachronal wave, which is required for the efficient pumping activity of ciliated epithelia [70,71]. Theoretical analyses suggested that hydrodynamic interactions between neighboring cilia cause the metachronal wave [72]. In addition, intracellular coupling of cilia, which is provided by cytoskeletal networks, mechanically link adjacent cilia and facilitate the formation of a metachronal wave [73,74,75,76]. Interestingly, with respect to the direction of the effective stroke, directions of metachronal waves vary among organs and animals and are likely related to the functions of organs [70,77]. An antiplectic wave is a metachronal wave that runs in the opposite direction to an effective stroke, and is suitable for carrying large objects. In contrast, a symplectic wave that runs in the same direction as an effective stroke, effectively generates fluid flow [70]. How the orientation of the metachronal wave is regulated is an open question in this field of research, although theoretical analyses suggest that several factors, including a spatial distribution of cilia, determine the pattern of a metachronal wave [72].

## 5. Roles of Cytoskeletons in Coordinating Cilia Movements

At the apical cortex of multi-ciliated cells, a meshwork of cytoskeleton, including microtubules and actin fibers, fill inter-cilia spaces [13,76,78,79,80,81,82]. As discussed earlier, various forms of evidence suggest that the connection between cilia and the cytoskeleton is key to coordinating cilia movements. In this section, we discuss how each cytoskeletal component interacts with cilia and coordinates their movements.

### 5.1. Microtubules

Imaging by transmission electron microscopy, as well as genetic and pharmacological analyses, suggests that the basal foot is tethered to microtubules [83,84] and that the link between the basal foot and microtubules is essential for orienting cilia [39,56,76,81,85,86,87]. At the apical cortex of multi-ciliated cells, two types of microtubules are associated with the basal foot. The first emanates from the tip of the basal foot and runs down to the center of the cell, while another runs around the basal foot in a horizontal plane [13,82]. In the *Xenopus* skin, microtubules interconnect neighboring basal bodies, thus enabling the local alignment of cilia orientation [76]. In addition, core PCP proteins control cilia orientation via microtubules as discussed earlier. Therefore, microtubules possibly play two roles in coordinating cilia orientation: one that aligns cilia at a local level between neighboring cilia, and another that aligns cilia along the tissue axis.

Gamma-tubulin is a well-known microtubule minus-end binding protein that nucleates and organizes microtubules. Immunogold labeling of gamma tubulin identified its localization at the tip of the basal foot [86]. The loss of galectin-3 reduced the amount of gamma tubulin at the basal foot, and disrupted the orientation of cilia. Furthermore, in mice lacking exons 6 and 7 of *Odf2*, the basal foot was lost and the orientation of cilia was weakly aligned in each multi-ciliated cell [85]. In both mutant mice, the apical microtubule network was disorganized. Based on those findings, the prevailing model would be that microtubules control the orientation of cilia via the basal foot while the basal foot provides a nucleation center for organizing the apical microtubule network.

Recently, another microtubule minus-end-binding protein, CAMSAP3, was shown to regulate the orientation of cilia [39,87,88]. In contrast to gamma-tubulin, CAMSAP3 was not detected at the tip of the basal foot and its localization varied among organs. In the trachea, CAMSAP3 was found at three regions along ciliary structures, namely regions more distal than the basal plate, the distal portion of the transition zone or at the basal plate, and the upper region of the basal body [87]. In the mouse oviduct, CAMSAP3 was localized at the basal body in the same direction as the basal foot, but was localized apically relative to the basal foot [39]. The loss of CAMSAP3 led to the misalignment of cilia orientation in the oviduct, trachea and nasal cavity, but not in the ventricle [89]. Furthermore, defects in the organization of the apical microtubule network were detected in the oviduct, but not in the trachea [39,87], suggesting that CAMSAP3 plays different roles in these organs, or that other redundant mechanisms are at work.

Although evidence suggested that microtubules control the orientation of cilia via the basal body, the underlying mechanisms remain elusive. Recent studies proposed a model in which pulling forces exerted by microtubules control the orientation of cilia [56,90]. In addition, a mathematical model incorporating polarity and self-organization activities of microtubules, reproduced the coordination of cilia orientation [57]. However, direct evidence supporting those models is still lacking. By connecting basal bodies with microtubules, gamma-tubulin and CAMSAP3 possibly enable the control of cilia orientation by microtubules. However, in *CAMSAP3* mutant cells, the structure of cilia is also affected. In the trachea, the central microtubule pair of the axoneme was lost and cilia showed abrogated movements [87]. In the oviduct, a small number of cyst-like protrusions were detected at the apical surface of multi-ciliated cells, and the protrusions occasionally contained microtubules or multiple axonemes [39]. Since the flow orients cilia, a disordered flow generated by the mutant cilia might exert a secondary effect on cilia orientation. Of note, even when the basal foot is lost in mice lacking exons 6 and 7 of *Odf2*, cilia orientation is partly coordinated, implying that a basal foot-independent mechanism also contributes to the alignment of cilia orientation [85].

### 5.2. Actin Filaments

In addition to microtubules, actin filaments form dense networks surrounding basal bodies [76,79,80,81,82]. However, they might play different roles in multi-ciliated cells. The roles of actin filaments in the apical migration of basal bodies during the differentiation of multi-ciliated cells have been thoroughly reviewed elsewhere [4,91]. Here, we focus on their roles in the coordination of cilia movements. In the skin of *Xenopus laevis*, two layers of an actin population, apical and subapical actin filaments, are located at the apical cortex of multi-ciliated cells. Subapical actin fibers connect the basal body of each cilium to the rootlet of neighboring cilia and this connection depends on FAK (Focal Adhesion Kinase), Paxillin, and Vinculin, which are well-known proteins that comprise focal adhesions [76,79]. When cells were treated with cytochalasin D at a concentration that specifically abolished subapical actin filaments, basal bodies were not uniformly distributed at the apical surface [76]. In addition, cilia orientation was not consistent over the entire cell surface, but was still locally coordinated between neighboring cilia. In contrast, nocodazole treatment did not affect the distribution of basal bodies, but prevented the alignment of cilia orientation, even at a local level [76]. Therefore, subapical actin filaments are required for the global coordination of cilia orientation and spacing of basal bodies in each cell, while microtubules locally align cilia orientation between neighboring cilia. Importantly, cytochalasin D treatment abolished a metachronal wave [76], suggesting that linkage of cilia via subapical actin fibers couples the timing of cilia movements. Furthermore, at the apical surface of *Xenopus* multi-ciliated cells, actin-rich cell protrusions form a labyrinth-like structure, which is referred to as a microridge-like structure. The rootlets of cilia are tethered to this microridge-like structure via subapical actin filaments, and this linkage contributes to the orientation of the rootlets [92]. It is still unclear whether such roles of actin filaments are conserved among animals, but layers of actin filaments at the apical cortex of multi-ciliated cells have been reported in various organs including mouse trachea and ventricle [81,93].

As discussed in the earlier section, PTEN, DVLs and active RhoA are localized at basal bodies and control both the ciliogenesis and cilia orientation. Since RhoA is required for the polymerization of actin fibers, these factors might contribute to the alignment of cilia orientation by organizing the apical actin networks. Again, one needs to pay attention to the role of these factors in orienting cilia; in cells lacking these factors, defects of ciliogenesis might exert a secondary effect on cilia orientation by affecting fluid flow.

## 6. Conclusions

In animal organs, cilia move mucus or oocytes, and generate fluid flow. To achieve such organ-level functions, the beating direction of cilia is coordinated at cellular and organ levels. Key machineries that orient cilia include the cytoskeleton and core PCP proteins, although the mechanisms of how these factors control cilia orientation remain unclear.

Multi-ciliated cells have several microtubule subpopulations, including apical microtubules running in parallel with the apical surface of the cell, as well as those emanating from basal bodies that run apico-basally. Nocodazole treatment of microtubules verified the involvement of microtubules in regulating the orientation of cilia [76,81], but it was unable to clarify the roles of each of microtubule subpopulation. Furthermore, the inhibition of microtubules affects intermediate filaments surrounding basal bodies [81]. To understand how the cytoskeleton controls the orientation of cilia, the roles of the microtubules and their subpopulation as well as actin filaments and intermediate filaments should be validated. For this purpose, each of them needs to be specifically manipulated in a spatio-temporally regulated manner. Recently established optogenetic tools that control cytoskeleton dynamics [94] would be useful for dissecting the roles of the cytoskeleton.

The mechanisms regulating the distribution of core PCP proteins remain elusive. Global cues in each ciliated organ and the mechanism by which they control the distribution of core PCP proteins need to be clarified. In addition, factors that act downstream of core PCP proteins are still not well understood. It seems that each core PCP protein functions differently, and that their roles vary in an organ-dependent manner. Since multi-ciliated cells move physically different objects in each organ, cells may use specific cassettes of core PCP proteins and downstream factors to generate functionally relevant multi-cilia. For example, in the oviduct and trachea, cilia are uniformly distributed, while in the ventricle, a patch of clustered cilia is formed. The position of the patch of clustered cilia is off-centered and biased along the tissue axis of the ventricle, and this is referred to as translational polarity. Genetic analysis suggested that translational polarity and rotational polarity were dependent on CELSR1 and CELSR3, respectively [41].

Recent advances in developmental biology highlight the roles of forces that sculpt an organism’s body. Multi-ciliated cells also take advantage of forces to coordinate the orientation of cilia and the timing of cilia beating. VANGL2 is required for the response of cilia to fluid flow, but the underlying mechanisms are still unclear. Since VANGL2 is localized both at cilia and cell boundaries, VANGL2 might play different roles at the two different places. Local degradation of core PCP proteins by using the light-induced protein degradation technique [95] would clarify their functions at cilia and cell boundaries. When a unicellular organism, *Paramecium*, collides with obstacles, the beating direction of cilia changes, and *Paramecium* moves away from the obstacles. In this process, the calcium ion acts as a messenger in response to the mechanical stimuli and changes the activity of dynein in cilia [96]. It would be interesting to observe whether VANGL2 and/or the calcium ion change their dynamics when artificial fluid is applied to multi-ciliated cells.

## Data Availability

Not applicable.
